# Antibiotic Delivery Strategies to Treat Skin Infections When Innate Antimicrobial Defense Fails

**DOI:** 10.3390/antibiotics9020056

**Published:** 2020-02-01

**Authors:** R. Smith, J. Russo, J. Fiegel, N. Brogden

**Affiliations:** 1 Department of Chemical and Biochemical Engineering, The University of Iowa, Iowa City, IA 52242, USA; riannon-smith@uiowa.edu (R.S.); jennifer-fiegel@uiowa.edu (J.F.); 2 Department of Pharmaceutical Sciences and Experimental Therapeutics, The University of Iowa, Iowa City, IA 52242, USA; jackson-russo@uiowa.edu; 3 Department of Dermatology, The University of Iowa, Iowa City, IA 52242, USA

**Keywords:** skin, wound, antimicrobial, antibiotic, infection, microbiome, bacteria, biofilm, topical

## Abstract

The epidermal skin barrier protects the body from a host of daily challenges, providing protection against mechanical insults and the absorption of chemicals and xenobiotics. In addition to the physical barrier, the epidermis also presents an innate defense against microbial overgrowth. This is achieved through the presence of a diverse collection of microorganisms on the skin (the “microbiota”) that maintain a delicate balance with the host and play a significant role in overall human health. When the skin is wounded, the local tissue with a compromised barrier can become colonized and ultimately infected if bacterial growth overcomes the host response. Wound infections present an immense burden in healthcare costs and decreased quality of life for patients, and treatment becomes increasingly important because of the negative impact that infection has on slowing the rate of wound healing. In this review, we discuss specific challenges of treating wound infections and the advances in drug delivery platforms and formulations that are under development to improve topical delivery of antimicrobial treatments.

## 1. Introduction

As the largest organ of the body, the skin presents a first line of defense against external microbes and pathogens. Skin wounds significantly compromise the integrity of the skin barrier, introducing a risk of local and systemic penetration of microbes through the compromised wound tissue. Skin wounds vary in type, presentation, depth, and comorbidities. The skin possesses excellent regenerative processes that permit rapid closure of skin tears and cuts. A wound that has not healed within 90 days is considered to be a chronic wound, and at this point, care and management become increasingly complicated [1]. Common types of chronic wounds include venous stasis ulcers, diabetic foot infections, and pressure ulcers (also known as “bed sores”). Wounds that have progressed to the chronic stage are a significant burden on the healthcare system, and it has been estimated that annually $25 billion is spent in the US alone on management of chronic wounds. This will likely continue to increase as the incidence of patients with obesity and diabetes rises, as both of these conditions present increased risk for development of chronic wounds [2]. 

Regardless of the source (e.g., burns, pressure ulcers, skin tears), skin wounds are painful and notoriously difficult to treat locally. Specific challenges with the use of topically applied treatments include a wet wound environment, local pain at the wound site, and the presence of inflammatory markers and enzymes within the wound tissue. Development of infection within the wound adds an additional challenge that increases the complexity of wound care practices because local infection delays healing of the wound [3]. Therefore, prevention and effective management of wound infections are of critical importance for patient safety and comfort. Here, we discuss challenges with current practices for treating wound infections and newer strategies being pursued in order to improve localized management.

## 2. Skin Properties and Wound Healing

The skin provides many protective and homeostatic functions to the body, including protection from external harmful stimuli such as chemicals, microbes, thermal damage, and radiation. The skin also contributes significantly to regulation of temperature and blood pressure. Owing to its critical role in maintaining homeostasis, the skin has a unique multi-layer structure distinct from any other organ in the body. 

**Figure 1:**
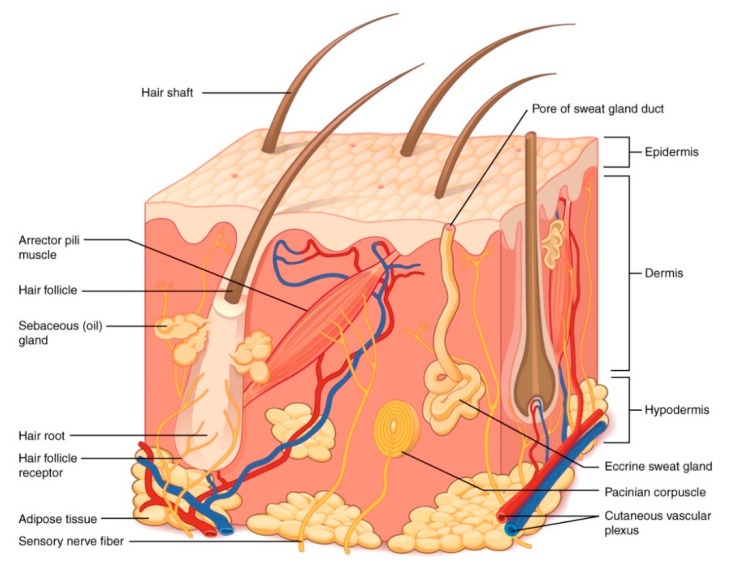
Structure of the skin. Illustration from Anatomy & Physiology, Connexions Web site. http://cnx.org/content/col11496/1.6/. This file is licensed under the Creative Commons Attribution 3.0 Unported license. No changes were made to the figure.

Starting from the outside in, the skin is composed primarily of four layers: the stratum corneum, the epidermis, the dermis, and the subcutaneous tissue (Figure 1). The stratum corneum is the outermost layer of the epidermis but often is discussed as a separate layer due to its unique properties. This layer of the skin is constantly exposed to the external environment and provides the primary permeability barrier function. The structure of the stratum corneum is often described as “brick and mortar”. The protein layer (cornified envelope) in keratinocytes serves as the bricks placed in between an intercellular lipid mortar of cholesterol, free fatty acids, and ceramides. The stratum corneum is no more than 10–15 µm thick over most of the body, with the exception of high friction surfaces such as the palms of the hands and the soles of the feet. 

Below the stratum corneum, the epidermal layer is ~100 µm thick. Melanocytes that produce melanin and give rise to skin pigmentation are found in the epidermis, as well as Langerhans cells that serve as the antigen-presenting cells in the skin’s immune response. Importantly, the epidermis is avascular and relies on the rich blood supply found in the dermis to provide oxygen and vital nutrients. The dermis is approximately 1000 µm thick and contains a rich capillary plexus that nurtures the skin. Sensor nerves for pain, temperature, and pressure are all found within the dermis, as is a rich lymphatic network. The dermis provides great structural support to the skin through the dense meshwork of collagen and elastin, amongst other structural fibers and ground substance. Skin appendages such as hair follicles and eccrine glands are contained here amongst the structural support. Beneath the dermis is the subcutaneous tissue containing blood vessels and adipocytes. The main role of this skin layer is to store lipids for energy (though it can serve as a reservoir of fatty acids that produce other bioactive lipid mediators) [4].

There are many specialized biochemical properties of the skin that contribute to the antimicrobial and the permeability barrier functions, though it is recognized that most of the skin’s defensive properties reside in the stratum corneum [5]. In its role as an antimicrobial barrier, the healthy unperturbed stratum corneum serves as virtually an absolute barrier to penetration of microbes. The lipids of the stratum corneum produce permeability, antioxidant, and antimicrobial barriers [6], and these bioactive lipids serve as a primary defensive barrier of the whole skin structure. This prevents microbes from reaching the viable skin layers below, which provide more desirable conditions for growth. The stratum corneum maintains an acidic pH of ~5.5, which is inhospitable to growth of most bacteria, in contrast to the higher physiologic pH in the epidermis and the dermis [5]. The stratum corneum sloughs off and is completely replaced approximately every two to four weeks, further contributing to an inhospitable environment for microbes.

In addition to the permeability barrier, another protection against infection is the presence of antimicrobial peptides (AMPs). These are generally synthesized in other layers of the epidermis, but the stratum corneum is the active site [6]. The AMPs, specifically β-defensins and cathelicidin, are generally small cationic polypeptides, and they play a large role in the skin’s innate defense mechanisms. They also have effects on inflammation, wound healing, and production of chemokines and cytokines [6,7]. 

The epidermis is inhabited by resident bacteria and fungi (commonly known as the microbiome or the microbiota). The majority of typical bacterial residents of the skin can be generally classified under four phyla: Proteobacteria, Firmicutes, Actinobacteria, and Bacteriodetes [8]. These same phyla are found in both the oral cavity and the gastrointestinal (GI) tract, though the ratios between the phyla are different. Under normal conditions, these microorganisms are in an intricate balance with a competent host immune system such that they do not cause infection. In fact, the microbe–host interaction plays a significant role in human health. Commensal microorganisms may inhibit growth of other pathogenic microbes through direct competition for resources when pathogens are introduced to the skin surface [8]. Commensal skin microbes can also influence host cells and thereby enhance cutaneous immunity. For instance, *Staphylococcus epidermidis* acts through both of these methods to increase skin defense through production of a serine protease that augments antimicrobial effects of the host antimicrobial peptide human β-defensin 2 and through inhibition of inflammatory cytokine production in a toll-like receptor driven process [8,9,10].

**Figure 2:**
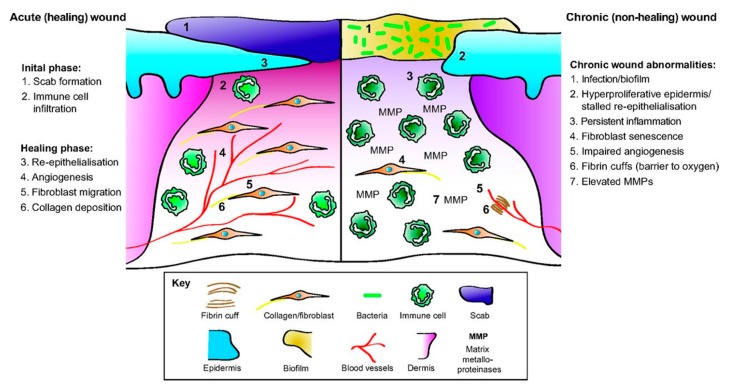
The cellular and the molecular differences between acute healing wounds and chronic non-healing wounds. The healing of acute wounds (left) initiates with a transient inflammatory response as granulation tissue is formed, which provides an environment suitable for the re-epithelialization required to complete repair. Chronic non-healing wounds (right) are often infected and exhibit persistent inflammation. By definition, re-epithelialization has stalled but is hyper-proliferative. Granulation tissue is sub-optimal with elevated matrix metalloproteinases (MMPs) present together with poor fibroblast and blood vessel infiltration. Fibrin cuffs can also be present that prevent the diffusion of oxygen through the wound, rendering it hypoxic. (Reprinted without changes from Nunan, et al. [13]. This is an open access article distributed under the Creative Commons Attribution License 4.0: https://creativecommons.org/licenses/by/4.0/.).

The skin has exceptional regenerative properties that allow most wounds to heal through a tightly regulated cascade of events. When the skin structure is compromised, the acute healing process begins. During normal wound healing (Figure 2), a transition from the initial inflammatory phase to the healing, or tissue regeneration, phase occurs typically within a few days. Tissue regeneration includes re-epithelialization and angiogenesis followed by tissue reorganization. Due to the inflammatory cascade and the presence of immune cells in early stages of healing, most pathogens that enter the open wound are readily eradicated so that skin infections do not occur every time the skin barrier is broken [2]. While chronic wounds are rarely seen in people who are generally otherwise healthy, they occur when a wound fails to progress through the normal cascade in a timely fashion. Chronic wounds typically get suspended in the inflammation stage and thus have shared characteristics such as excessive proinflammatory cytokines, reactive oxygen species, proteases, and persistent infection [11]. These wounds can remain open for years, enhancing the chances of infection [12].

## 3. Bacterial Infection of Wounds

Because skin is the first defense against many pathogenic bacteria, a wound, especially a large wound, can present an opening for easy access into the body [14,15]. Opportunistic pathogens, those that become pathogenic upon some perturbation of the host (e.g., disease, wound, immunodeficiency, or aging), can grow and multiply once they enter the body by colonizing on nutrient-rich substances such as necrotic or hypoxic tissue [16,17]. Colonizing microbes come from the environment (in the air or the soil, introduced through traumatic injury, hospital personnel, or instruments) or the body’s natural flora (on the surrounding skin or mucous membranes). If the pathogenic microbes invade viable tissue, invasive infection can occur [15]. A compromised immune system or underlying medical condition can lead to chronic wounds that remain open longer, resulting in higher microbial exposure and potential colonization [18].

The immune system combats infection by stimulating inflammation in both the injured tissue and the body (Figure 2, Initial phase) [15]. The keratinocytes in the skin release cytokines, antimicrobial peptides, and antimicrobial proteins that eradicate pathogens, while neutrophils, macrophages, and mast cells release inflammatory cytokines and growth factors critical for the wound repair process [12]. The immune cells can further kill and remove pathogens and injury debris. These defenses effectively eradicate many microbes that colonize minor wounds, enabling normal wounds to progress through the wound healing stages [12]. As the wound heals, there is less opportunity for pathogens to colonize the wound. However, for wounds that are slow to heal (such as burns and chronic wounds), that have altered cytokine release patterns (such as in diabetes mellitus), or that form biofilms, infections inhibit the wound healing process [19]. 

Various Gram-positive, Gram-negative, aerobic, and anaerobic bacteria as well as different species of fungi have been isolated from skin infections [17,20]. The most common bacterial pathogens implicated in wound infections include *Staphylococcus aureus*, *Enterococcus spp.*, *Streptococcus pyogenes*, *Escherichia coli,* and *Pseudomonas aeruginosa* [15,21,22]. Other bacteria include *Klebsiella pneumoniae, Acinetobacter spp., and Enterobacter spp.* Healthcare settings can introduce nosocomial infections, including *E. coli, S. aureus, K. pneumoniae*, and methicillin-resistant *S. aureus* (MRSA) infections [23,24,25,26]. The role of fungi, particularly *Candida* and *Aspergillus* species, in wounds is being increasingly recognized but has not been sufficiently studied [15]. Fungi typically colonize a wound later than Gram-positive or Gram-negative bacteria [27]. The delay in colonization has been attributed to the use of broad-spectrum antimicrobials during the first two weeks after injury [15]. The specific pathogens that colonize a wound depend on a variety of host factors (such as wound etiology; site, size, and depth of the wound; immune status; comorbidities; age; inadequate nutrition) as well as pathogen factors (such as the microbial load and the virulence of the microorganisms) [17,28].

Once a wound is colonized, pathogenic microbes continue to interact with the host in ways that impact health and healing. At a critical colonization level, the healing process is delayed, and an increase in pain may occur [29,30]. Thus, bacteria levels must be reduced to enable the healing process to resume. Many pathogenic bacteria produce virulence factors that damage tissues and, if not treated, can introduce systemic effects. *P. aeruginosa* produces adhesins, exotoxin A, hemolysins, and proteases that allow it to adhere to tissue, take in nutrients, kill immune cells, and destroy tissue. *S. aureus,* the cause of most skin and soft tissue infections, produces hemolysins, leukocidins, and superantigens, which allow the microbes to adhere to and destroy cells and tissues [15,31]. Infections that penetrate deep into the wound can result in systemic effects, such as sepsis [22,31]. Pathogens may also form biofilms, colonies of microbes that attach to the wound surface and form a protective extracellular matrix, making them more resistant against antimicrobial therapies.

## 4. Current Clinical Care Practices to Combat Skin Infections

Treatment of wounds involves maintaining a clean, moist environment, managing pain, and addressing underlying comorbidities contributing to the wound (poor glycemic control, adequate nutrition, etc.). Systemic medical treatments are often required for management of pain and infection, thus introducing complexities of systemic adverse events, drug interactions, and inadequate local drug concentrations within the wound itself. A myriad of topical therapies and dressings are available for use, ranging from (and not limited to) sterile saline to hydrogels, iodine solutions, honey, hypochlorous acid, and collagenase. These are often used in combination with commercial dressings including sterile gauze, alginates, hydrocolloids, and others. One of the most important functions of wound dressings is to keep the wound covered, moist, and protected from contamination.

Wound treatment is often complicated by the increased susceptibility to infection, and infection is one of the most important single causes of delayed healing. There are four interrelated variables that contribute to therapeutic efficacy when treating an infected, chronic wound: (1) local tissue concentrations of antimicrobials at the infection site; (2) microbial flora of the site; (3) presence of ischemia or necrotic tissue that impairs drug distribution; and (4) antimicrobial resistance [32]. Early treatment of infected skin wounds includes debridement of necrotic tissue, antibiotic application, and covering the wound [33]. Historically, antimicrobials have been administered both topically and systemically (oral or intravenous route) for treating chronic wound infections [32]. The range of systemic drugs that have been used in this capacity is quite large, spanning nearly every major class of antibiotics including (but not limited to) aminoglycosides, beta-lactams, lincosamides, macrolides, and quinolones [32]. However, this introduces many difficult clinical challenges. There is insufficient evidence to support “routine” use of systemic antibiotics in some types of chronic wounds, including diabetic ulcers and presence of a biofilm, as large variations in tissue concentrations of antibiotics occur [34,35,36]. Owing to the challenges of systemic antibiotics, they should be viewed as part of a multifaceted approach and reserved for treating spreading local infections or systemic infection. 

Topical (local) antimicrobial treatments are often used for wound infections. Topical treatments provide antimicrobial activity at the source of the infection and can have fewer systemic side effects [37]. Topical therapy provides both high and sustained concentrations of antimicrobial at the wound site, and overall a more limited amount of antimicrobial is needed. Local administration can limit the potential for systemic absorption of antibiotics, thus reducing antibiotic resistance. Compared to the broad array of antibiotics that are delivered systemically, the list of topical antibiotics is much smaller, including: bacitracin, neomycin, polymyxin, mupirocin, and fusidic agents. Antiseptics commonly used for topical treatment of wound infections can be divided into four broad classes: emulsifiers (chlorhexidine, octenidine, polyhexamethylene biguanide, benzalkonium salts), oxidizers (hydrogen peroxide, dilute hyperchlorite preparations, iodine compounds), acids (acetic acid, honey, boric acid), and heavy metals (silver compounds, bismuth compounds, copper, and mercury) [32,38]. Not all of these agents are commonly used for treatment of skin wounds, as some are used primarily to reduce microbial burden on the skin surface prophylactically (chlorhexidine, octenidine, povidone iodine), while others are often used for irrigation and wound cleansing capacities (chlorhexidine). Below, we specifically discuss topical uses of silver ions and iodophor (iodine) compounds, as both have extensive clinical data describing their use in wound care [38]. 

### 4.1. Wound Treatments Using Silver Ions

Silver is the most commonly used topical antimicrobial in the United States [38]. Silver ions work by binding the peptidoglycans of bacterial cell membranes being taken up by the cell and interfering with a variety of cellular functions [39]. Because of the diverse killing mechanisms, there is very little reported bacterial resistance to silver ions, and their delivery has been explored using a variety of delivery vehicles. Commercially available products include liquid solutions of AgNO_3_, ointments of silver sulfadiazine (Silvadene, Pfizer, Inc), and a large array of impregnated dressings [40]. The AgNO_3_ comes in solutions with concentrations varying from 0.5% to 50%, though 0.5% is the standard concentration [38]. Silver sulfadiazine cream, which was first introduced in the 1960s, is used to prevent bacterial colonization of wounds such as burns [41]. This product is a combination of sulfadiazine (a sulfa antibiotic) and silver ions; the product typically is used in a 1% cream [38]. AgNO_3_ and silver sulfadiazine both maintain moist environments that promote wound healing while also preventing the dressings from adhering to the wound and causing pain on application. More recently, new ways of delivering silver ions to wound sites have been developed, including dressings, hydrogels, and foams that contain silver ions [42].

While silver ion products themselves show good antimicrobial activity, the cytotoxic effect of silver ions extends beyond bacterial cells. The cytotoxic effect of silver ions on human cells may therefore present problems in the long-term wound healing process. A 2012 study found that silver ions were cytotoxic to human mesenchymal stem cells at a concentration of 2.5 ppm [43]. This concentration is approximately the same as the concentration needed to kill bacteria, meaning that the use of antimicrobial silver ions may also be detrimental to the wound healing process. The use of silver in treating wounds is certainly effective in preventing bacterial colonization in the early stages of wound treatment, but because of the lack of selectivity in their cytotoxic mechanisms, their use should be limited to avoid impairing the healing process.

### 4.2. Wound Treatments Using Molecular Iodine

Iodine has been used as an antimicrobial agent in wound care for hundreds of years. Iodine achieves its antimicrobial action by penetrating microorganisms and attacking key protein groups (in particular, cysteine and methionine), fatty acids, and nucleotides [44]. It has proven effective against a broad spectrum of bacteria, including many antibiotic-resistant species [45,46,47,48]. Unlike silver, which must be delivered in its ionized form to show antibacterial activity, iodine’s antimicrobial effect is only exerted when it is in its molecular form [20]. Traditionally, iodine was simply formulated as a solution of iodine molecules in water or alcohol, but such a solution results in undesirable staining of the wound site and hyperalgesia [49]. Additionally, a solution of iodine is unstable and exists as an equilibrium of several iodine species. Researchers have developed alternative formulation techniques to provide iodine molecules to wound sites while minimizing adverse effects.

To avoid the problems presented by solutions of molecular iodine, contemporary antimicrobial iodine products used in wound care typically use iodine carrying systems, or “iodophors” [50]. One of the most commonly used iodophors is povidone-iodine (PVP-I), in which iodine is tightly bound to povidone, a carrier polymer, and released over time to exert its antimicrobial effect [51]. PVP-I complexes are used for their antimicrobial activity in wounds as solutions, ointments, swabs, and aerosol sprays [52]. PVP-I is most commonly known as Betadine, available in an ointment and liquid formulation, consisting of polyvinylpyrrolidone and 9–12% elemental iodine [38].

Using a similar principle, iodine has also been formulated in ointment, powder, paste dressing, and gel forms as a complex with cadexomer, a dextrin-based complexing agent [53]. Additionally, cadexomer-iodine bandages have been used as wound dressings that provide antibacterial activity through the release of iodine molecules over time into the wound site, while the cadexomer polymer provides an absorptive bandage to remove exudate [54]. This agent is commonly used for chronic venous leg ulcers [38,55]. The majority of studies examining the effect of iodine treatments on wound healing indicate that both PVP-I and cadexomer-iodine treatments have been observed to improve the rate of wound healing in patients with chronic wounds [56]. 

The main concern regarding the use of iodine as an antimicrobial agent is adverse effects resulting from systemic exposure. A 1998 study indicated that use of cadexomer iodine paste in wound treatment was correlated with a significantly higher serum iodine concentration than was observed in groups treated with dressings without iodine [57]. Within a subset of the population, including children and newborns, iodine has been known to induce hypothyroidism [58]. Other potential side effects include acidosis, dermatitis, and renal failure; some of these rare side effects limit the routine use of iodine products in burn wounds [38]. 

### 4.3. Wound Treatments Using Honey

Naturally occurring treatments have been used for a variety of conditions since ancient times, where products such as honey have been used to help with sore throats, colds, and topical infections [59]. Honey has natural antimicrobial and antioxidant properties, which can limit infection and help epithelialization in wounded tissue [60]. Honey gets its antimicrobial activity from a combination of high osmolarity and the production of hydrogen peroxide from the glucose oxidase in honey [3,27]. It also keeps the wound bed moist, which promotes healing. While honey is not a standard of care in the United States, there are medically-certified honeys available for wound treatment in Europe and Australia [61]. The properties of honey depend on many factors, including plants, season, bees, and geographic location [3,62]. These differences suggest that particular honey types are needed to maximize its use in medicine. Medeiros et al. used honey from *Melipona scutellaris*, a wild bee in Northeast Brazil, as an antimicrobial agent against an MRSA wound infection in rats [63]. The honey treatment was compared with a saline solution treatment, with the infected wounds treated with honey having fewer counts of *S. aureus*. Their research also showed that the wounds treated with honey had higher counts of healing parameters, including collagen, fibroblasts, and leukocytes, and thus had better healing properties than saline alone [63]. Honey can also be mixed with other compounds to enhance the antioxidant activity and promote good skin structure recovery. Abderrahim et al. developed a mixture from Euphorbia honey and *Allium sativum L*. (garlic) extract, which exhibited microbial inhibition of *S. aureus*, *E. coli*, *P. aeruginosa*, and *C. albicans* [60]. The honey and extract mixture further reduced the time needed for epithelialization of the wound compared to betadine solution or honey by itself.

## 5. Drug Delivery Challenges of Infected Wounds

Despite the clear advantages of using topical therapy for treating wound infections, wound environments present a number of significant challenges for delivering antimicrobial agents to the locally infected site. Logistical issues, pain, and heterogeneity of the wound environment contribute to the clinical challenges. Additional specific challenges include achieving sufficient local antimicrobial concentrations, the presence of biofilms, simultaneous treatment of multiple pathogens in a wound, altered local pH and inflammation of the wounded tissue environment, and maintaining an environment that promotes healing. While these variables present challenges for treatment, they also provide opportunities for targeted treatment using drug delivery systems that can respond to specific stimuli within the wound.

### 5.1. Mechanical Challenges

Some problems with topically treating an infected wound relate simply to mechanical and logistical application issues. Application of traditional creams and ointments can be very painful for a patient. A hallmark of chronic wounds and burn wounds is the sensitivity of the wound area, and the manual application of spreading a topical product within an already painful site can be excruciating. Treatment of open wounds necessitates a sterile formulation, which introduces a manufacturing challenge. Accurate and reproducible dosing can be difficult, and multidose containers have the potential to become contaminated (especially if frequent reapplication is necessary). If a wound is of significant size, some systemic absorption of topically applied antimicrobials may still occur and thereby introduce some of the common challenges and toxicity often seen with systemic administration.

### 5.2. Wound Environment

For optimal healing, chronic wounds and burns need to stay moist during the treatment and healing process, as this results in faster epithelialization and enhanced angiogenesis and collagen synthesis than a dry environment [64,65]. Additionally, a moist wound environment allows faster breakdown of dead tissue and fibrin, resulting in less scarring [66,67]. Finally, perhaps counterintuitively, maintenance of wound moisture has been observed to correlate to a significantly lower infection rate than keeping the wound site dry [68]. During the healing process, the moist occluded environment promotes a lower pH, making the wound overall less hospitable for microbes [69]. Keeping the wound moist is also a balancing act because moisture promotes bacterial and fungal growth, and too much moisture can cause tissue break down in the wound bed [70].

The compromised skin barrier and the wet environment of wounds present notable challenges for development of a topical formulation that can adhere to the tissue. While maintaining a locally moist environment, drug delivery from semisolid dosage forms such as creams and gels is difficult because high moisture content can affect mucoadhesion of the product, stability, and extent/rate of drug delivery from the dosage form. 

Prolonged inflammation in chronic wounds is associated with a high burden of reactive oxygen species, and wound exudate contains salts and proteases that may change the wound microenvironment and ultimately impact the delivery vehicle, the drug itself, or the effectiveness of treatment. The pH in chronic wounds is alkaline (as opposed to the slightly acidic pH of normal skin) [71], which can cause changes in the performance of topical antibiotics and antiseptics. Active ingredients may change in ionization status under fluctuating pH, and some antibiotics such as gentamicin may have enhanced activity because of decreased transport into the bacteria under more acidic environments [72]. Increased pH has also been found to increase effectiveness of antibiotic activity against *S. aureus*, whereas fluoroquinolones have enhanced activity under more acidic conditions [73,74]. The minimum inhibitory concentration (MIC) of some bacteria may be lower at higher pH, potentially increasing antimicrobial efficacy [75]. Activity of antiseptics such as silver and iodine under alkaline pH have not been well documented, though it is likely that performance is affected. Solubility of metal ions generally decreases with lower pH, and thus the solubility and the bioavailability will be reduced under conditions of high pH. Biochemical stability of ionic compounds such as silver will also be affected, thereby decreasing bioavailability and reducing efficacy (generally at pH above 6.0) [76].

### 5.3. Achieving Local Concentrations of Antimicrobials

With any antimicrobial treatment, the appropriate antimicrobial agents must be maintained at the appropriate concentrations to inhibit growth and eradicate all microbes present at the site of infection, as sub-inhibitory concentrations of antimicrobial agents drive genomic changes that lead to antibiotic resistance and pathogen persistence [77]. As multiple pathogens may be present in a wound site, broad spectrum antimicrobials or multiple antimicrobial drugs must be delivered to the site of infection. When systemic antibiotics are used to treat wound infections, high serum levels are required to achieve appropriate concentrations in the target tissue. This can result in significant adverse effects, including ototoxicity and nephrotoxicity [78]. Topical treatments can be applied directly to the wound and diffuse into surrounding tissue without causing systemic effects. Thus, the antimicrobial activity may be directed specifically toward the colonizing bacteria within the wound (this is especially important for antiseptic agents that rely only on a local mechanism of action) [32]. There are many challenges that come with this approach, however. If topical treatments are applied too late, at the wrong concentration, or do not affect all of the microbes present, bacteria may develop resistance. The moist environment required for optimal wound healing and suppression of microbial growth can change the properties of topically applied gels and creams applied to the site. Thus, use of these delivery systems is not very effective because they absorb fluid and become more mobile [40], reducing local action of the applied drugs. Therefore, delivery systems must be designed with these challenges in mind.

### 5.4. Biofilm Mechanisms that Resist Treatment

Biofilms are microbial communities of tightly packed cell aggregates within a secreted, sticky extracellular matrix [14,79]. As planktonic cells are often orders of magnitude easier to treat than the biofilms, it is critical to treat infections early, preferably before biofilms form or reach maturity [80,81]. The biofilm matrix is composed primarily of polysaccharides, nucleic acids, lipids, and proteins that form a physical barrier of protection and also contribute to antibiotic resistance [82]. Certain matrix components, including DNA, bacterial surface proteins, and quorum sensing molecules, induce an inflammatory host response [83,84]. The continuous activation of the immune response due to these molecules can result in damage to the host tissue and delay wound healing. Biofilms further disperse bacteria into the surrounding environment. As dispersed cells can have higher rates of phenotypic variation, the infection can spread with new local acute infections developing that are more resistant than the original infection [80,85]. Biofilm formation is particularly problematic with chronic wounds, as prolonged exposure to the environment increases their chance of becoming established. This has been shown with clinical samples, where biofilms were identified in 60% of chronic wound specimens but only in 6% of acute wound specimens [86].

Biofilms present several major challenges to topical drug delivery, including the protective barrier created by the extracellular matrix, the slow growth of microorganisms within the biofilm, and the mutations in the microbes that allow the acquisition of genes that afford drug resistance [83,87]. The extracellular matrix forms a physical barrier protecting the microbes against immune cells and antimicrobials. In addition, its chemical constituents are involved in adhesion, aggregation, and redox activities that can further limit the activity of immune cells and antimicrobials within the biofilm [84]. The biofilm community consists of both planktonic and biofilm bacteria, which differ in their susceptibility to antimicrobials. The biofilm bacteria are in a state of slow metabolic activity due to nutrient and oxygen limitations in the biofilm. As the majority of antimicrobials are effective against metabolically active microbes, targeting processes such as cell division, DNA replication, and protein synthesis, this limits the effectiveness of antimicrobial treatments and may promote antibiotic resistance [80,81]. Mutational resistance mechanisms include up-regulation of efflux pumps, membrane and target modifications, and enzyme production. By any of these mechanisms, resistant bacteria can survive and continue to grow in the presence of an antimicrobial, thus resulting in chronic infections. As wound biofilms are typically polymicrobial, it is likely that combination treatments containing antimicrobial agents with varied killing mechanisms are needed for treatment and to manage the drug resistant microbes [2,80,88]. Beyond the standard antibiotic therapies, non-conventional strategies may provide better treatment options for wound biofilms [89].

## 6. Alternative Antimicrobial Treatments

To overcome known problems with current treatments for skin infections, alternative strategies are being researched and developed with the aim to provide better treatment options for open wound infections. Here, we highlight systems under development along with their benefits and remaining known challenges (summarized in Table 1). The following section is separated into three subsections (hydrogels, nanofibers, and nanoparticles), though it is important to note that there are many systems under development that combine these areas. Hydrogels loaded with antimicrobial nanoparticles, hydrogels, and nanoparticles that use naturally-occurring polymers, and nanoparticles made using natural products are examples of products all under development. The combinations of several of these systems are used to improve antimicrobial efficacy, improve mechanical properties, and alter drug release. Another area, though not discussed in detail in this review, of alternative treatments comes from natural products. Natural products can overcome some of the key issues with the widespread use of synthetic antimicrobial agents by reducing side effects and the occurrence of drug resistant pathogens [60]. Natural components including aloe, cellulose, natural plant extracts, and fish skin collagen have been studied as chronic and burn wound dressings [90,91,92,93,94,95,96]. 

### 6.1. Hydrogels

Hydrogels are promising wound dressings due to their high water content, flexibility, and biocompatibility [126]. Some of the most common hydrogel components include polyvinyl alcohol, polyethylene glycol, poloxamers, and carboxymethyl cellulose (described in Table 2). Other hydrogels have been made using artificial bolalipids, gelatin, collagen, sericin, and other polymer matrices [98,121,124,126,127]. While there are several polymer types that are commonly used for the fabrication of hydrogels, hydrogel properties can be altered by copolymerization, cross-linking, or combining multiple polymers into the network. The hydrophilicity of hydrogels is due to the presence of hydrophilic groups on the polymer chains, such as alcohols, amides, amines, carboxylic acids, and sulfonic acids. When the polymer network is placed in water, physical and chemical crosslinking prevents the polymer from dissolving in water [128]. Depending on the polymer composition, hydrogels can be formulated for biodegradation, possibly eliminating the need for removal [129,130]. As wound dressings, they cover the wound and protect the exposed area, maintain a moist wound environment, absorb wound exudate, and can provide antimicrobial properties [40,90,131,132,133,134]. Because of the variety of microorganisms found in infected wounds, hydrogels that can deliver antibiotics or are inherently antimicrobial may provide critical wound treatment [15,22]. Various antimicrobial agents have been loaded into hydrogels for topical wound delivery, including gold and silver nanoparticles, fusidic acid, antimicrobial peptides, probiotic organisms, and other synthetic antimicrobial compounds [135,136,137,138,139,140]. Hydrogels can provide slow release of the antimicrobial agents over extended periods of time, which could decrease dressing change frequency and limit exposure to sub-inhibitory concentrations of antimicrobials.

Polyvinyl alcohol (PVA), made by hydrolyzing polyvinyl acetate, is a hydrophilic polymer that absorbs moisture and maintains a moist wound environment [40,110]. The polymer properties depend on the degree of hydrolysis, concentration, temperature, and freezing cycles, which enables tunability of properties for various applications [110,144]. PVA has been blended and modified using synthetic and natural polymers in order to achieve desirable hydrogels [129]. The literature of PVA-based hydrogels for delivery of antimicrobials for wound infection is extensive, with studies describing hydrogel systems of minocycline [159], gentamicin [160], and nitrofurazone [161], among others. This continues to be a very rich area of study, with more recent work detailing complex delivery systems (nanofibrous mats, films, and sponges) for delivery of silver [162], zinc nanoparticles [163], benzalkonium bromide [164], and cationic antimicrobial peptides [113]. The variety of delivery systems and formulations that have been developed using PVA for antimicrobial wound dressings underscores the versatility of this material for treatment of wound infections.

Polyethylene glycol (PEG) is a hydrophilic polymer available in a wide range of different molecular weights based on the number of repeating units of −(O−CH_2_−CH_2_)−. It is nontoxic and approved by the Food and Drug Administration (FDA) [151,165]. Due to its biocompatibility and its versatility, PEG is typically combined or copolymerized with other polymer systems to achieve desired properties such as solubility [166], tunable drug release rate [167], and resistance to bioadhesion [168]. PEG is used in wound dressings since it can retain water and keep the wound environment moist [169]. Common uses of PEG in wound dressing applications include PEGylation of chitosan [170] and copolymerization of PEG with other polymers to make thermosensitive hydrogels [146]. PEG can also be used to make other polymers, including polyethylene glycol diacrylate (PEGDA), a common polymer crosslinker used in hydrogels, and polyethylene glycol methacrylate (PEGMA), a photopolymerizable polymer that can be incorporated to enable the entrapment and the release of hydrophobic drugs [171,172]. Extensive reports are available in the literature describing PEGylation for antimicrobial delivery, including PEGylated polymers, nanoparticles, nanofibers, and fibrin-based wound dressings [173,174,175].

Poloxamers (sold under the trade names Kolliphor, Pluronics, and Synperonics) are triblock copolymers with polyethylene oxide and polypropylene oxide segments [135,136,176,177,178]. They have generated interest because they form thermoreversible hydrogels, meaning they are liquid at lower temperatures and transition to a gel at higher temperatures. The critical transition temperature is based on polymer type, molecular weight, concentration, and ratio of the polyethyene oxide and the polypropylene oxide segments [179]. When applied as cool liquids, thermoreversible hydrogels can spread over irregular areas before gelation is complete, thereby providing better wound coverage. Poloxamer 407 in particular has been studied in great detail for drug delivery and biomedical applications, likely because, at polymer concentrations ranging from 15 – 20%, it will form a gel at or near physiologic temperatures [179]. Drug release from poloxamer hydrogels is based on rate of drug diffusion out of the gel matrix and erosion/dissolution of the matrix itself [147]. Desired drug release, degradation, adhesion, and mechanical properties of poloxamers can be achieved by incorporating other hydrogel components or crosslinking the network [179,180]. Arafa et al. added hydroxypropyl methylcellulose to Pluronic^®^ F127 to improve gel strength and tune the release rate of antimicrobial gold nanoparticles [136]. The combination of hydroxypropyl methylcellulose and Pluronic led to a stronger gel, a lower gelation temperature, and reduced the burst-like release of gold nanoparticles compared to the Pluronic gel alone [136]. In a permeation study, both formulations showed the presence of the gold nanoparticles at the diffusion site for up to five hours. The developed gels cleared bacteria from a wound earlier than silver sulfadiazine [136].

Carboxymethyl cellulose (CMC) is a natural, water-soluble polysaccharide made of a cellulose backbone containing a large number of carboxymethyl groups [151]. It is present in some commercially used dressings, such as Aquacel^®^ from ConvaTec and Urgotul^®^ SSD from Urgo [181]. When applied to a wound, CMC can absorb wound exudate and form a gel. In fact, a class of hydrogels known as superabsorbent hydrogels (SAP) based on celullose derivatives have been developed in order to absorb large quantities of water or physiological solutions [151]. CMC dressings are typically not stiff, provide a moist wound environment, and can be removed [1]. Cellulose derivatives also carry the benefits of low cost and can be formulated into a variety of preparations including films, wafers, and gels. CMC can be blended and polymerized with other materials, including polyethylene glycol (PEG), to form hydrogel matrices [151]. CMC also exists in salt form (NaCMC) to form intermolecular crosslinks in a blended hydrogel matrix, whereas native CMC tends to form intramolecular links [182]. CMC has also been combined with zinc oxide nanoparticles to form an antimicrobial and pH-sensitive hydrogel [155] and silver nanoparticles in a hydrogel that is effective against Gram-negative and Gram-positive bacterial strains [183]. Recent work has also demonstrated that NaCMC can effectively deliver antimicrobials to treat wound infection when developed as a wafer [153]. 

Hyaluronic acid is a polysaccharide naturally found in the skin. This compound helps reduce inflammation, is biocompatible and biodegradable, stimulates fibroblast and keratinocyte proliferation, and is proangiogenic [184,185,186]. There has been a recent upswing in commercial products that include hyaluronic acid, including face creams and lotions, because of its hydration effect on the skin. While hyaluronic acid is naturally occurring in humans, for widespread application as a treatment, it is either produced from bacteria or harvested from rooster combs [187]. While it does not have any inherent antimicrobial properties, it can improve wound healing, thus limiting wound exposure to pathogens [40,188]. It can also serve as the starting material for hydrogel formation and modify the release of antimicrobial agents [178,185,189,190]. Contardi et al. combined polyvinylpyrrolidone and hyaluronic acid into a bilayer membrane loaded with ciprofloxacin as a treatment for infection and to assist in remodeling of the wounded tissue [189]. The presence of the hyaluronic acid controlled the release of ciprofloxacin for five days, whereas a faster, burst-like release was observed without hyaluronic acid. The developed membrane was effective against *S. aureus*, *E. coli*, and *P. aeruginosa* [189]. It remains to be seen whether this system will promote faster wound healing and exhibit antimicrobial effects in vivo; however, they did report that the membrane did not present any complications or toxic effects in an in vivo mouse model with a full-thickness excisional wound.

While many hydrogels are loaded with an antimicrobial agent, hydrogels that possess antimicrobial properties on their own have also been developed [140]. These antimicrobial hydrogels can be made from naturally occurring substances, including chitosan or peptide-based hydrogels [191], or by synthesizing polymers with antimicrobial groups [91,192,193]. They are promising treatment options because they require a lower concentration of antimicrobial agent to achieve the same effects and provide a different mechanism of killing microbes compared to antibiotics and antiseptics. The reduction in antimicrobial concentration could lead to lower toxicity and limit antimicrobial resistance [25]. Chen et al. developed an antimicrobial hydrogel by synthesizing a multi-arm thiolated PEG crosslinked with silver nitrate [192]. The network is antimicrobial because it gradually releases silver ions. The hydrogel also exhibits self-healing behavior due to the sulfur-silver coordination bond. Against *S. aureus*, the hydrogel both with and without an angiogenic drug showed antimicrobial activity. In a wound healing model, the hydrogel loaded with the angiogenic drug exhibited increased healing rate, lowering the available infection time of the wound [192]. Irwansyah et al. developed an antimicrobial hydrogel of 9-fluoroenylmethoxycarbonyl-modified oligopeptides with phenylalanine (gelator) and leucine (antimicrobial). The developed hydrogel was active against Gram-positive bacteria by disrupting the bacterial cell walls and membranes [194]. Other antimicrobial hydrogels being developed include peptide-based hydrogels and polymer-based hydrogels containing poly(hexamethylene) biguanide hydrochloride (PHMB) hydrogels, polycarbonate containing quaternary ammonium groups, or zwitterionic polymers [40,156,195]. 

Chitosan, deacetylated chitin found in insect exoskeletons, shrimp, crab, and some fungi, is another naturally occurring polysaccharide used extensively in applications in the medical and the food industries [193]. It is biocompatible and biodegradable and can be produced with various molecular weights ranging from 300 to over 1000 kD [196]. When chitosan chains are crosslinked with physical or chemical crosslinks, they can form a hydrogel [197]. Because chitosan is positively charged under normal physiologic conditions, it can form ionic interactions with constituents of the bacterial cell wall, interfering with the cell’s barrier function and sensitizing the microbes to antimicrobial compounds [198]. Because of this activity, there has been a steep incline in publications using chitosan and derivatives of chitosan for antimicrobial applications [193]. Ma et al. developed a chitosan wound dressing with ZnO based N-halamine nanoparticles and tested the dressing against *S. aureus* and *E. coli* [118]. Chitosan exhibited mild antibacterial activity against the two bacterial strains, but the nanoparticle-loaded chitosan dressings reduced bacterial loads by up to 99.9% and 88% for *S. aureus* and *E. coli*, respectively. While chitosan exhibits some level of antimicrobial properties, alone it was not enough to eradicate the bacteria in this study. However, the chitosan increased the bacterial sensitivity to the antimicrobial nanoparticles, resulting in an enhanced antimicrobial effect when given in combination [118].

### 6.2. Nanoscale Materials

Nano-scale therapies, including nanofibers and nanoparticles, are being developed as drug delivery systems for antimicrobial agents [103]. Nanofibers can be woven into mats that provide coverage similar to other wound dressings. Nanomaterials, due to their size, are well-suited for penetration and delivery of antimicrobials into biological structures, including tissues and biofilms [199]. These systems are made from a wide range of materials, most commonly of polymers, metals, and metal oxides [103,109,115,200,201]. While promising, these therapies must be closely monitored for biocompatibility and cytotoxic effects from the high concentration of material in the tissue.

Nanofibers are fibers with nanometer-scale diameter that are typically made using electrospinning, self-assembly, or solution blowing [1,105,202]. Nanofibers have a high surface to volume ratio and porosity, which can increase drug loading, transport, and cell attachment [196,200,202]. Nanofiber scaffolds mimic the structure of the extracellular matrix, which can help with cell proliferation, thereby promoting re-epithelialization and wound closure [1,203,204]. The porosity of nanofiber matrices aids the adsorption of wound exudate, the exchange of nutrients, and provides coverage against bacterial attachment [204,205]. Nanofibers can be synthesized from a wide range of material, including biodegradable and non-biodegradable polymers, naturally occurring polymers, and composite materials [203]. Altinbasak et al. developed a reusable nanofiber mat made of poly(acrylic acid) and reduced graphene-oxide fibers loaded with ampicillin and cefepime, which released drug when near-infrared light heated the mat [103]. The nanofiber mats had very slow diffusion when not light stimulated, thereby showing good retention of the antibiotics (good for shelf life). Both antibiotics released simultaneously with light at concentrations above the minimum inhibitory concentration for *E. coli*, *S. epidermidis*, and *S. aureus*. Su et al. developed a nanofiber mat containing a core of pluronic F127 and an electrosprayed shell made from poly(ε-caprolactone) (PCL) and an antimicrobial peptide [110]. The electrosprayed mat generated an initial burst release, which may be favorable in wound applications to achieve an effective concentration rapidly and then sustain the release over time. The electrosprayed mat eliminated *P. aeruginosa* bacterial colonies within three days of daily dressing changes in an in vivo full-thickness excisional wound treated initially by debridement [110]. These recent studies suggest excellent wound treatment using nanofibers; however, their use in clinical practice is limited due to toxicity, large-scale production, and ability to control capacity and functionality [206].

Nanoparticles are of significant interest to the field for their ability to penetrate deep into damaged skin and affect microbial growth. Properly designed, nanoparticle therapies enhance antibiotic effects by delivering high concentrations of antimicrobial agent to the application site while reducing adverse effects such as the development of drug-resistant microbes [165,199]. Because silver is widely used in topical antimicrobial products, its use in nanoparticle form has received substantial attention, demonstrating antimicrobial activity [109,115,181]. Other metallic nanoparticles such as gold, zinc, and copper also show good antimicrobial properties [110,136,207]. However, several metal nanoparticles, including silver, copper oxide, and zinc oxide, have exhibited toxic effects in the body [25]. Therefore, care must be taken to ensure that nanoparticle size, surface chemistry, dissolution, and dissolution products are optimized to provide good antimicrobial effects but limit toxicity. As an alternative, polymeric nanoparticles can be designed for greater biocompatibility and to sustain the release of antimicrobial agents over longer periods of time [25]. For example, Hasan et al. developed two poly(D,L-lactide-co-glycolide) (PLGA) nanoparticles, positively or negatively charged, loaded with the antimicrobial agent clindamycin [201]. Both particles exhibited an initial burst release, enabling killing of bacteria initially present in the local environment and then sustained release over two days, enabling maintenance of an inhibitory concentration. The positively charged particles exhibited slightly faster release and attachment to MRSA cell walls, which reduced the bacterial load and promoted healing in a MRSA-infected wound. Nanoparticles, whether metal or polymeric, have exhibited antimicrobial activity against a wide variety of organisms. They can be used as a stand-alone treatment or incorporated into other systems to prolong antimicrobial release. Overall, nanoparticles are a versatile treatment option (with a wide variety of formulations) that maintain high antimicrobial concentrations in the wound bed but likely require another vehicle to keep them in the applied area.

## 7. Conclusions

Treatment of wound infections has been a long-standing clinical challenge complicated by the presence of biofilms, multi-drug resistant microbes, and a heterogenous wound environment. Systemic antibiotics are not always appropriate for treatment of wound infections, but we have yet to encounter an approach that overcomes all of the challenges of topically applying antimicrobials to a wound. Advances in hydrogels, nanomaterials, and natural products may offer expanded treatment options for wound infections through products that offer better drug release and degradation profiles, improved adhesion, and superior mechanical properties within the wound. These formulations also offer more flexibility in materials and compounds that can be used, which will further provide a better array of treatment possibilities for patients suffering from wound infections. As the magnitude of this challenge continues to grow, it is imperative that research efforts in this area continue to explore novel delivery systems for topical antibiotic delivery.

## Figures and Tables

**Table 1:** Description of key advantages and challenges associated with the delivery approaches in various stages of development for topical delivery of antimicrobials to wounds.

Treatment Type	Development Level	Advantages	Challenges
**Hydrogels** [67,97,98,99,100,101]	Several formulations available in clinical use; additional formulations in research and animal testing phases	Keep wound environment moist; absorb wound exudate; allow oxygen transmission to the wound; cooling effect; can achieve sustained release; wide variety of natural and synthetic polymer options; forms to irregular wound areas	Poor mechanical properties; moist environment may support fungal infection and bacterial colonization
**Nanofibers** [19,30,93,102,103,104,105,106,107,108,109,110,111,112,113,114]	Rapidly growing area in research applications; many compositions in research and animal testing phases	Various release profiles can be obtained; absorbs wound exudate; structure aids cell proliferation; high mechanical performance; wide variety of natural and synthetic polymer options; large surface area to volume ratio; good oxygen exchange with environment	Relatively new material; difficult to choose the appropriate material to fabricate nanofibers of desired size with desired technique; adhesion to wound can negatively affect healing upon removal; significant concerns still exist for controlling drug delivery, functionality, toxicity, and large-scale production
**Nanoparticles** [19,115,116,117,118,119,120,121]	Many are FDA approved, though not for treatment of wound infection; many nanoparticle products in clinical trials	Deep penetration in wounds; biofilm penetration; can provide high antimicrobial concentration at the site; sustained release profiles can be achieved	Fabrication standards/quality; clearance; toxicity of certain metal nanoparticles; containing nanoparticles to delivery site
**Natural Products** [63,92,94,95,96,102,107,111,112,121,122,123,124,125]	Have been used for many years; possible synergistic effects with other treatment strategies	Decreased toxicity effects; less known antibiotic resistance	Varying efficacy depending on source; lack of purification standards; difficult to determine efficacy based on lack of standards for evaluation

FDA = Food and Drug Administration.

**Table 2:** Description of key advantages and challenges associated with specific polymers used in hydrogel products.

Hydrogel Agent	Advantages	Challenges	Modifications
PVA* [67,98,110,121,124,132,141,142,143,144]	Biocompatible; antifouling properties; hydrophilic; biodegradable	No intrinsic antimicrobial activity; low mechanical strength prior to freeze-thaw process	Crosslinked with natural and synthetic polymers; nanoparticle incorporation; freeze–thaw process
PEG* [97,100,119,145,146]	Vast clinical experience from use; biocompatible; many molecular weights for different systems; tunable drug delivery profile	No intrinsic antimicrobial activity	Thiolated and crosslinked; blend with natural or synthetic polymers; often used as a hydrogel crosslinker or to provide better properties to other polymers
Poloxamers* [130,135,136,138,147,148,149]	Thermoreversible; tunable gelling and release properties; can be applied as a cool solution that is soothing to wounds and spreads well	No intrinsic antimicrobial activity; degrades and dissolves easily	HPMC to tune release and viscosity; can use mixtures of multiple poloxamers to tune release
Carboxymethyl cellulose* [150,151,152,153,154,155]	Water soluble; natural product; biocompatible and biodegradable; inexpensive; can be formulated into multiple types of wound dressings	Forms intramolecular crosslinks (not intermolecular); hydrocolloid by itself; no intrinsic antimicrobial activity	Salt form for intermolecular crosslink; blend or polymerize with other compounds
Chitosan * [97,125,129,156,157,158]	Has inherent antimicrobial activity; natural product; biocompatible; can be fabricated into multiple types of wound dressings	Higher cost; can be more difficult to handle compared to other hydrogels	Molecular weight and degree of deacetylation; PEGylation; crosslinking; combined with other hydrogels such as PVA

PVA = polyvinyl alcohol, PEG = polyethylene glycol, HPMC = hydroxypropyl methylcellulose. * Found in FDA approved products.

## References

[1] Saghazadeh S., Rinoldi C., Schot M., Kashaf S.S., Sharifi F., Jalilian E., Nuutila K., Giatsidis G., Mostafalu P., Derakhshandeh H. (2018). Drug delivery systems and materials for wound healing applications. Adv. Drug Del. Rev..

[2] Sen C.K. (2019). Human wounds and its burden: An updated compendium of estimates. Adv. Wound Care.

[3] Punjataewakupt A., Napavichayanun S., Aramwit P. (2019). The downside of antimicrobial agents for wound healing. Eur. J. Clin. Microbiol. Infect. Dis..

[4] Kendall A.C., Nicolaou A. (2013). Bioactive lipid mediators in skin inflammation and immunity. Prog. Lipid Res..

[5] Elias P.M. (2007). The skin barrier as an innate immune element. Semin. Immunopathol..

[6] Lee S.H., Jeong S.K., Ahn S.K. (2006). An update of the defensive barrier function of skin. Yonsei Med. J..

[7] Aberg K.M., Man M.-Q., Gallo R.L., Ganz T., Crumrine D., Brown B.E., Choi E.-H., Kim D.-K., Schröder J.M., Feingold K.R. (2008). Co-Regulation and Interdependence of the Mammalian Epidermal Permeability and Antimicrobial Barriers. J. Invest. Dermatol..

[8] Sanford J.A., Gallo R.L. (2013). Functions of the skin microbiota in health and disease. Semin. Immunol..

[9] Iwase T., Uehara Y., Shinji H., Tajima A., Seo H., Takada K., Agata T., Mizunoe Y. (2010). Staphylococcus epidermidis Esp inhibits Staphylococcus aureus biofilm formation and nasal colonization. Nature.

[10] Lai Y., Cogen A.L., Radek K.A., Park H.J., MacLeod D.T., Leichtle A., Ryan A.F., Di Nardo A., Gallo R.L. (2010). Activation of TLR2 by a Small Molecule Produced by Staphylococcus epidermidis Increases Antimicrobial Defense against Bacterial Skin Infections. J. Invest. Dermatol..

[11] Frykberg R.G., Banks J. (2015). Challenges in the Treatment of Chronic Wounds. Adv. Wound Care.

[12] MacLeod A.S., Mansbridge J.N. (2016). The Innate Immune System in Acute and Chronic Wounds. Adv. Wound Care.

[13] Nunan R., Harding K.G., Martin P. (2014). Clinical challenges of chronic wounds: Searching for an optimal animal model to recapitulate their complexity. Dis. Model. Mech..

[14] Wysocki A.B. (2002). Evaluating and managing open skin wounds: Colonization versus infection. AACN Clin. Issues.

[15] Church D., Elsayed S., Reid O., Winston B., Lindsay R. (2006). Burn wound infections. Clin. Microbiol. Rev..

[16] Grice E.A., Segre J.A. (2012). Interaction of the Microbiome with the Innate Immune Response in Chronic Wounds.

[17] Bowler P.G., Duerden B.I., Armstrong D.G. (2001). Wound microbiology and associated approaches to wound management. Clin. Microbiol. Rev..

[18] Han G., Ceilley R. (2017). Chronic wound healing: A review of current management and treatments. Adv. Ther..

[19] Sandri G., Miele D., Faccendini A., Bonferoni M.C., Rossi S., Grisoli P., Taglietti A., Ruggeri M., Bruni G., Vigani B. (2019). Chitosan/Glycosaminoglycan Scaffolds: The Role of Silver Nanoparticles to Control Microbial Infections in Wound Healing. Polymers.

[20] Bradshaw C.E. (2011). An in vitro comparison of the antimicrobial activity of honey, iodine and silver wound dressings. Biosci Horizons.

[21] Garms B.C., Borges F.A., de Barros N.R., Marcelino M.Y., Leite M.N., Del Arco M.C., Salvador S.L.D., Pegorin G.S., Oliveira K.S.M., Frade M.A.C. (2019). Novel polymeric dressing to the treatment of infected chronic wound. Appl. Microbiol. Biotechnol..

[22] Norbury W., Herndon D.N., Tanksley J., Jeschke M.G., Finnerty C.C. (2016). Infection in burns. Surg. Infect. (Larchmt.).

[23] Erol S., Altoparlak U., Akcay M.N., Celebi F., Parlak M. (2004). Changes of microbial flora and wound colonization in burned patients. Burns.

[24] Otter J.A., Yezli S., Salkeld J.A.G., French G.L. (2013). Evidence that contaminated surfaces contribute to the transmission of hospital pathogens and an overview of strategies to address contaminated surfaces in hospital settings. Am. J. Infect. Control.

[25] Hemeg H.A. (2017). Nanomaterials for alternative antibacterial therapy. Int. J. Nanomedicine.

[26] Iyer S., Jones D.H. (2004). Community-acquired methicillin-resistant Staphylococcus aureus skin infection: A retrospective analysis of clinical presentation and treatment of a local outbreak. J. Am. Acad. Dermatol..

[27] Dai T., Huang Y.Y., Sharma S.K., Hashmi J.T., Kurup D.B., Hamblin M.R. (2010). Topical antimicrobials for burn wound infections. Recent Pat. Anti-infect. Drug Discov..

[28] Casadevall A., Pirofski L.-A. (2018). What Is a Host? Attributes of Individual Susceptibility. Infect. Immun..

[29] Aljghami M.E., Saboor S., Amini-Nik S. (2019). Emerging Innovative Wound Dressings. Ann. Biomed. Eng..

[30] Dhand C., Venkatesh M., Barathi V.A., Harini S., Bairagi S., Goh Tze Leng E., Muruganandham N., Low K.Z.W., Fazil M.H.U.T., Loh X.J. (2017). Bio-inspired crosslinking and matrix-drug interactions for advanced wound dressings with long-term antimicrobial activity. Biomaterials.

[31] Gonzalez C.D., Ledo C., Cela E., Stella I., Xu C.L., Ojeda D.S., Frenette P.S., Gomez M.I. (2019). The good side of inflammation: Staphylococcus aureus proteins SpA and Sbi contribute to proper abscess formation and wound healing during skin and soft tissue infections. Biochim. Biophys. Acta-Mol. Basis Dis..

[32] Leaper D., Assadian O., Edmiston C.E. (2015). Approach to chronic wound infections. Br. J. Dermatol..

[33] Stone R., Natesan S., Kowalczewski C.J., Mangum L.H., Clay N.E., Clohessy R.M., Carlsson A.H., Tassin D.H., Chan R.K., Rizzo J.A. (2018). Advancements in regenerative strategies through the continuum of burn care. Front. Pharmacol..

[34] O’Meara S., Cullum N., Majid M., Sheldon T. (2000). Systematic reviews of wound care management: (3) antimicrobial agents for chronic wounds; (4) diabetic foot ulceration. Health Technol. Assess..

[35] Seabrook G.R., Edmiston C.E., Schmitt D.D., Krepel C., Bandyk D.F., Towne J.B. (1992). Comparison of serum and tissue antibiotic levels in diabetes-related food infections. Surgery.

[36] Edmiston C.E., Goheen M.P., Seabrook G.R., Johnson C.P., Lewis B.D., Brown K.R., Towne J.B. (2006). Impact of selective antimicrobial agents on staphylococcal adherence to biomedical devices. Am. J. Surg..

[37] Honeywell-Nguyen P.L., Bouwstra J.A. (2005). Vesicles as a tool for transdermal and dermal delivery. Drug Discov. Today Technol..

[38] Cambiaso-Daniel J., Boukovalas S., Bitz G.H., Branski L.K., Herndon D.N., Culnan D.M. (2018). Topical Antimicrobials in Burn Care: Part 1-Topical Antiseptics. Ann. Plast. Surg..

[39] Kramer A., Dissemond J., Kim S., Willy C., Mayer D., Papke R., Tuchmann F., Assadian O. (2018). Consensus on Wound Antisepsis: Update 2018. Skin Pharmacol. Physiol..

[40] Boateng J., Catanzano O. (2015). Advanced therapeutic dressings for effective wound healing-a review. J. Pharm. Sci..

[41] Fox C.L. (1968). Silver Sulfadiazine—A New Topical Therapy for Pseudomonas in Burns: Therapy of Pseudomonas Infection in Burns. Arch. Surg..

[42] Walker M., Parsons D. (2012). The biological fate of silver ions following the use of silver-containing wound care products—a review. Int. Wound J..

[43] Greulich C., Braun D., Peetsch A., Diendorf J., Siebers B., Epple M., Köller M. (2012). The toxic effect of silver ions and silver nanoparticles towards bacteria and human cells occurs in the same concentration range. RSC Advances.

[44] McDonnell G., Russell A.D. (1999). Antiseptics and disinfectants: Activity, action, and resistance. Clin. Microbiol. Rev..

[45] Traoré O., Fayard S.F., Laveran H. (1996). An in-vitro evaluation of the activity of povidone-iodine against nosocomial bacterial strains. J. Hosp. Infect..

[46] Giacometti A., Cirioni O., Greganti G., Fineo A., Ghiselli R., Del Prete M., Mocchegiani F., Fileni B., Caselli F., Petrelli E. (2002). Antiseptic Compounds Still Active Against Bacterial Strains Isolated from Surgical Wound Infections Despite Increasing Antibiotic Resistance. Eur. J. Clin. Microbiol. Infect. Dis..

[47] McLure A.R., Gordon J. (1992). In–vitro evaluation of povidone–iodine and chlorhexidine against methicillin–resistant Staphylococcus aureus. J. Hosp. Infect..

[48] Mertz P.M., Oliveira-Gandia M.F., Davis S.C. (1999). The Evaluation of a Cadexomer Iodine Wound Dressing on Methicillin Resistant Staphylococcus Aureus (MRSA) in Acute Wounds. Dermatol. Surg..

[49] Lawrence C.A., Carpenter C.M., Naylor-Foote A.W.C. (1957). Iodophors as Disinfectants, Calif. J. Am. Pharm. Assoc. (Sci. ed.).

[50] Capriotti K., Capriotti J.A. (2012). Topical iodophor preparations: Chemistry, microbiology, and clinical utility. Dermatol. Online J..

[51] Bigliardi P.L., Alsagoff S.A.L., El-Kafrawi H.Y., Pyon J.-K., Wa C.T.C., Villa M.A. (2017). Povidone iodine in wound healing: A review of current concepts and practices. Int. J. Surg..

[52] Ripa S., Bruno R., Reder R., Casillis R., Roth R. (2002). Clinical applications of povidone–iodine as a topical antimicrobial. Handbook of Topical Antimicrobials Industrial Applications, Industrial Applications in Consumer Products and Pharmaceuticals.

[53] Raju R., Kethavath S.N., Sangavarapu S.M., Kanjarla P. (2019). Efficacy of Cadexomer Iodine in the Treatment of Chronic Ulcers: A Randomized, Multicenter, Controlled Trial. Wounds.

[54] Holloway G.A., Johansen K.H., Barnes R.W., Pierce G.E. (1989). Multicenter trial of cadexomer iodine to treat venous stasis ulcer. West. J. Med..

[55] Skog E., Arnesjo B., Troeng T., Gjores J.E., Bergljung L., Gundersen J., Hallbook T., Hessman Y., Hillstrom L., Mansson T. (1983). A randomized trial comparing cadexomer iodine and standard treatment in the out–patient management of chronic venous ulcers. Br. J. Dermatol..

[56] Angel D., Morey P., Storer J., Mwipatayi B. (2008). The great debate over iodine in wound care continues: A review of the literature. Wound Pract. Res..

[57] Hansson M.D., Carita P., Group T.C.I.S. (1998). The effects of cadexomer iodine paste in the treatment of venous leg ulcers compared with hydrocolloid dressing and paraffin gauze dressing. Int. J. Dermatol..

[58] Leung A.M., Braverman L.E. (2014). Consequences of excess iodine. Nat. Rev. Endocrinol..

[59] Kumar K.P., Bhowmik B., Chiranjib C., Biswajit B., Chandira M.R. (2010). Medicinal uses and health benefits of Honey: An Overview. J. Chem. Pharm. Res..

[60] Abderrahim L.A., Taibi K., Abderrahim N.A., Boussaid M., Rios-Navarro C., Ruiz-Sauri A. (2019). Euphorbia honey and garlic: Biological activity and burn wound recovery. Burns.

[61] Yaghoobi R., Kazerouni A., Kazerouni O. (2013). Evidence for Clinical Use of Honey in Wound Healing as an Anti–bacterial, Anti–inflammatory Anti–oxidant and Anti–viral Agent: A Review. Jundishapur. J. Nat. Pharm. Prod..

[62] El Sohaimy S.A., Masry S.H.D., Shehata M.G. (2015). Physicochemical characteristics of honey from different origins. Ann. Agric. Sci..

[63] Medeiros V.d.F.L.P., Azevedo Í.M., Rêgo A.C.M., Egito E.S.T.d., Araújo-Filho I., Medeiros A.C. (2016). Antibacterial properties and healing effects of Melipona scutellaris honey in MRSA–infected wounds of rats. Acta. Cir. Bras..

[64] Winter G.D. (1962). Formation of the Scab and the Rate of Epithelization of Superficial Wounds in the Skin of the Young Domestic Pig. Nature.

[65] Hinman C.D., Maibach H. (1963). Effect of Air Exposure and Occlusion on Experimental Human Skin Wounds. Nature.

[66] Junker J.P.E., Kamel R.A., Caterson E.J., Eriksson E. (2013). Clinical Impact Upon Wound Healing and Inflammation in Moist, Wet, and Dry Environments. Adv. Wound Care.

[67] Jaiswal M., Koul V., Dinda A.K. (2016). In vitro and in vivo investigational studies of a nanocomposite–hydrogel–based dressing with a silver–coated chitosan wafer for full–thickness skin wounds. J. Appl. Polym. Sci..

[68] Hutchinson J.J., McGuckin M. (1990). Occlusive dressings: A microbiologic and clinical review. Am. J. Infect. Control.

[69] Kruse C.R., Nuutila K., Lee C.C.Y., Kiwanuka E., Singh M., Caterson E.J., Eriksson E., Sørensen J.A. (2015). The external microenvironment of healing skin wounds. Wound Repair Regen..

[70] Zeng R., Lin C., Lin Z., Chen H., Lu W., Lin C., Li H.J.C., Research T. (2018). Approaches to cutaneous wound healing: Basics and future directions. Cell Tissue Res..

[71] Jones E.M., Cochrane C.A., Percival S.L. (2015). The Effect of pH on the Extracellular Matrix and Biofilms. Adv. Wound Care.

[72] Eisenberg E.S., Mandel L.J., Kaback H.R., Miller M.H. (1984). Quantitative association between electrical potential across the cytoplasmic membrane and early gentamicin uptake and killing in Staphylococcus aureus. J. Bacteriol..

[73] Lamp K.C., Rybak M.J., Bailey E.M., Kaatz G.W. (1992). In vitro pharmacodynamic effects of concentration, pH, and growth phase on serum bactericidal activities of daptomycin and vancomycin. Antimicrob. Agents Chemother..

[74] Emrich N.-C., Heisig A., Stubbings W., Labischinski H., Heisig P. (2010). Antibacterial activity of finafloxacin under different pH conditions against isogenic strains of Escherichia coli expressing combinations of defined mechanisms of fluoroquinolone resistance. J. Antimicrob. Chemother..

[75] Garrod L.P., Waterworth P.M. (1956). Behaviour in vitro of some new antistaphylococcal antibiotics. Br. Med. J..

[76] Percival S.L., McCarty S., Hunt J.A., Woods E.J. (2014). The effects of pH on wound healing, biofilms, and antimicrobial efficacy. Wound Repair Regen..

[77] Penesyan A., Nagy S.S., Kjelleberg S., Gillings M.R., Paulsen I.T. (2019). Rapid microevolution of biofilm cells in response to antibiotics. NPJ Biofilms and Microbiomes.

[78] DeCoster T.A., Bozorgnia S. (2008). Antibiotic Beads. JAAOS—J. Am. Acad. Orthop. Surg..

[79] Namgoong S., Jung S.Y., Han S.K., Kim A.R., Dhong E.S. (2019). Clinical experience with surgical debridement and simultaneous meshed skin grafts in treating biofilm–associated infection: An exploratory retrospective pilot study. J. Plast. Surg. Hand Surg..

[80] Wu H., Moser C., Wang H.-Z., Hoiby N., Song Z.-J. (2015). Strategies for combating bacterial biofilm infections. Int. J. Oral Sci..

[81] Hengzhuang W., Wu H., Ciofu O., Song Z., Høiby N. (2011). Pharmacokinetics/pharmacodynamics of colistin and imipenem on mucoid and nonmucoid Pseudomonas aeruginosa biofilms. Antimicrob. Agents Chemother..

[82] Kirker K.R., James G.A. (2017). In vitro studies evaluating the effects of biofilms on wound–healing cells: A review. APMIS.

[83] Bahamondez-Canas T.F., Heersema L.A., Smyth H.D.C. (2019). Current Status of In Vitro Models and Assays for Susceptibility Testing for Wound Biofilm Infections. Biomedicines.

[84] Zhao G., Usui M.L., Lippman S.I., James G.A., Stewart P.S., Fleckman P., Olerud J.E. (2013). Biofilms and Inflammation in Chronic Wounds. Adv. Wound Care.

[85] Koh K.S., Lam K.W., Alhede M., Queck S.Y., Labbate M., Kjelleberg S., Rice S.A. (2007). Phenotypic diversification and adaptation of Serratia marcescens MG1 biofilm–derived morphotypes. J. Bacteriol..

[86] James G.A., Swogger E., Wolcott R., Pulcini E.d., Secor P., Sestrich J., Costerton J.W., Stewart P.S. (2008). Biofilms in chronic wounds. Wound Repair Regen..

[87] Davies D. (2003). Understanding biofilm resistance to antibacterial agents. Nat. Rev. Drug Discov..

[88] Bertesteanu S., Triaridis S., Stankovic M., Lazar V., Chifiriuc M.C., Vlad M., Grigore R. (2014). Polymicrobial wound infections: Pathophysiology and current therapeutic approaches. Int. J. Pharm..

[89] Kadam S., Shai S., Shahane A., Kaushik K.S. (2019). Recent Advances in Non–Conventional Antimicrobial Approaches for Chronic Wound Biofilms: Have We Found the ’Chink in the Armor?. Biomedicines..

[90] Boonmak N., Niyompanich J., Chuysinuan P., Niamlang P., Ekabutr P., Supaphol P. (2018). Preparation of mangosteen extract–loaded poly(vinyl acetate) for use as an antibacterial spray–on dressing. J. Drug Deliv. Sci. Technol..

[91] Sahiner N., Sagbas S., Sahiner M., Silan C., Aktas N., Turk M. (2016). Biocompatible and biodegradable poly (Tannic Acid) hydrogel with antimicrobial and antioxidant properties. Int. J. Biol. Macromol..

[92] Xi J.Q., Wu Q.W., Xu Z.L., Wang Y.Q., Zhu B.B., Fan L., Gao L.Z. (2018). Aloe–Emodin/Carbon Nanoparticle Hybrid Gels with Light–Induced and Long–Term Antibacterial Activity. Acs Biomater. Sci. Eng..

[93] Gomaa S.F., Madkour T.M., Moghannem S., El-Sherbiny I.M. (2017). New polylactic acid/ cellulose acetate–based antimicrobial interactive single dose nanofibrous wound dressing mats. Int. J. Biol. Macromol..

[94] Sadri M., Karimi-Nazari E., Hosseini H., Emamgholi A. (2016). New chitosan/poly(ethylene oxide)/thyme nanofiber prepared by electrospinning method for antimicrobial wound dressing. J. Nanostruct..

[95] Ramanauskien K., Stelmakiene A., Majien D. (2015). Assessment of Lemon Balm (Melissa officinalis L.) Hydrogels: Quality and Bioactivity in Skin Cells. Evid. Based Complement. Alternat. Med..

[96] Lukac P., Hartinger J.M., Mlcek M., Popkova M., Suchy T., Supova M., Zavora J., Adamkova V., Benakova H., Slanar O. (2019). A novel gentamicin–releasing wound dressing prepared from freshwater fish Cyprinus carpio collagen cross–linked with carbodiimide. J. Bioact. Compatible Polym..

[97] Anjum S., Arora A., Alam M.S., Gupta B. (2016). Development of antimicrobial and scar preventive chitosan hydrogel wound dressings. Int. J. Pharm..

[98] Imtiaz N., Niazi M.B.K., Fasim F., Khan B.A., Bano S.A., Shah G.M., Badshah M., Menaa F., Uzair B. (2019). Fabrication of an original transparent PVA/gelatin hydrogel: In vitro antimicrobial activity against skin pathogens. Int. J. Polym. Sci..

[99] Babavalian H., Latifi A.M., Shokrgozar M.A., Bonakdar S., Mohammadi S., Moosazadeh Moghaddam M. (2015). Analysis of Healing Effect of Alginate Sulfate Hydrogel Dressing Containing Antimicrobial Peptide on Wound Infection Caused by Methicillin–Resistant Staphylococcus aureus. Jundishapur J. Microbiol..

[100] Chen H., Cheng R.Y., Zhao X., Zhang Y.H., Tam A., Yan Y.F., Shen H.K., Zhang Y.S., Qi J., Feng Y. (2019). An injectable self–healing coordinative hydrogel with antibacterial and angiogenic properties for diabetic skin wound repair. Npg. Asia Materials.

[101] Murray R.Z., West Z.E., Cowin A.J., Farrugia B.L. (2019). Development and use of biomaterials as wound healing therapies. Burns & Trauma.

[102] Abdelgawad A.M., Hudson S.M., Rojas O.J. (2014). Antimicrobial wound dressing nanofiber mats from multicomponent (chitosan/silver–NPs/polyvinyl alcohol) systems. Carbohydr. Polym..

[103] Altinbasak I., Jijie R., Barras A., Golba B., Sanyal R., Bouckaert J., Drider D., Bilyy R., Dumych T., Paryzhak S. (2018). Reduced Graphene–Oxide–Embedded Polymeric Nanofiber Mats: An "On–Demand" Photothermally Triggered Antibiotic Release Platform. Acs. Appl. Mater. Interfaces.

[104] Bakhsheshi-Rad H.R., Ismail A.F., Aziz M., Akbari M., Hadisi Z., Daroonparvar M., Chen X.B. (2019). Antibacterial activity and in vivo wound healing evaluation of polycaprolactone–gelatin methacryloyl–cephalexin electrospun nanofibrous. Mater. Lett..

[105] Chen W., Yang S., Li S., Lang J.C., Mao C., Kroll P., Tang L., Dong H. (2019). Self–Assembled Peptide Nanofibers Display Natural Antimicrobial Peptides to Selectively Kill Bacteria without Compromising Cytocompatibility. ACS. Appl. Mater. Interfaces.

[106] Feng Y.B., Wang Q., He M., Zhang X., Liu X.L., Zhao C.S. (2019). Antibiofouling zwitterionic gradational membranes with moisture retention capability and sustained antimicrobial property for chronic wound infection and skin regeneration. Biomacromolecules.

[107] Hassiba A.J., El Zowalaty M.E., Webster T.J., Abdullah A.M., Nasrallah G.K., Khalil K.A., Luyt A.S., Elzatahry A.A. (2017). Synthesis, characterization, and antimicrobial properties of novel double layer nanocomposite electrospun fibers for wound dressing applications. Int. J. Nanomedicine.

[108] Liao N., Unnithan A.R., Joshi M.K., Tiwari A.P., Hong S.T., Park C.-H., Kim C.S. (2015). Electrospun bioactive poly (ɛ–caprolactone)–cellulose acetate–dextran antibacterial composite mats for wound dressing applications. Colloids Surf. Physicochem. Eng. Aspects.

[109] Nhi T.T., Minh H.H., Nam T.M.P., Thien D.B.T., Hoai N.T.T., Phuoc T.V., Thai D.M., Hai N.D., Toi V.V., Hiep N.T. (2018). Optimization and characterization of electrospun polycaprolactone coated with gelatin–silver nanoparticles for wound healing application. Mater. Sci. Eng. C–Mater. Biol. Appl..

[110] Paduraru A., Ghitulica C., Trusca R., Surdu V.A., Neacsu I.A., Holban A.M., Birca A.C., Iordache F., Vasile B.S. (2019). Antimicrobial wound dressings as potential materials for skin tissue regeneration. Materials.

[111] Ramalingam R., Dhand C., Leung C.M., Ong S.T., Annamalai S.K., Kamruddin M., Verma N.K., Ramakrishna S., Lakshminarayanan R., Arunachalam K.D. (2019). Antimicrobial properties and biocompatibility of electrospun poly–ε–caprolactone fibrous mats containing Gymnema sylvestre leaf extract. Mater. Sci. Eng. C..

[112] Sarhan W.A., Azzazy H.M.E., El-Sherbiny I.M. (2016). Honey/Chitosan Nanofiber Wound Dressing Enriched with Allium sativum and Cleome droserifolia: Enhanced Antimicrobial and Wound Healing Activity. ACS Appl. Mater. Interfaces.

[113] Sebe I., Ostorhazi E., Fekete A., Kovacs K.N., Zelko R., Kovalszky I., Li W., Wade J.D., Szabo D., Otvos L. (2016). Polyvinyl alcohol nanofiber formulation of the designer antimicrobial peptide APO sterilizes Acinetobacter baumannii–infected skin wounds in mice. Amino Acids.

[114] Sedghi R., Shaabani A. (2016). Electrospun biocompatible core/shell polymer–free core structure nanofibers with superior antimicrobial potency against multi drug resistance organisms. Polymer.

[115] Chhibber S., Gondil V.S., Singla L., Kumar M., Chhibber T., Sharma G., Sharma R.K., Wangoo N., Katare O.P. (2019). Effective Topical Delivery of H–AgNPs for Eradication of Klebsiella pneumoniae–Induced Burn Wound Infection. AAPS. PharmSciTech..

[116] Farag R.K., Labena A., Fakhry S.H., Safwat G., Diab A., Atta A.M. (2019). Antimicrobial activity of hybrids terpolymers based on magnetite hydrogel nanocomposites. Materials.

[117] Hua J.S., Teng P., Zou Y.Y., Zhang C., Shen X.J., Cai J.F., Hu Y. (2018). Small antimicrobial agents encapsulated in poly(epsilon–caprolactone)–poly (ethylene glycol) nanoparticles for treatment of S. aureus–infected wounds. J. Nanopart. Res..

[118] Ma W., Li L., Lin X.H., Wang Y.F., Ren X.H., Huang T.S. (2019). Novel ZnO/N–halamine–mediated multifunctional dressings as quick antibacterial agent for biomedical applications. ACS Appl. Mater. Interfaces.

[119] Rabiee T., Yeganeh H., Gharibi R. (2019). Antimicrobial wound dressings with high mechanical conformability prepared through thiol–yne click photopolymerization reaction. Biomed. Mater..

[120] Refat M.S., Elsabawy K.M., Alhadhrami A., Almalki A.S.A., El-Sayed M.Y., Hassan R.F. (2018). Development of medical drugs: Synthesis and in vitro bio–evaluations of nanomedicinal zinc–penicillins polymeric hydrogel membranes for wound skin dressing by new chemical technology. J. Mol. Liq..

[121] Rinehart S.J., Campbell T.D., Burke K.J., Garcia B., Mlynarski A., Brain S.J., Truffa J.M., Rago J., Chura W.E., Keleher J.J. (2016). Synthesis and characterization of a chitosan/PVA antimicrobial hydrogel nanocomposite for responsive wound management materials. J. Microb. Biochem. Technol..

[122] Fayemi O.E., Ekennia A.C., Katata-Seru L., Ebokaiwe A.P., Ijomone O.M., Onwudiwe D.C., Ebenso E.E. (2018). Antimicrobial and Wound Healing Properties of Polyacrylonitrile–Moringa Extract Nanofibers. ACS Omega.

[123] Mebert A.M., Alvarez G.S., Peroni R., Illoul C., Helary C., Coradin T., Desimone M.F. (2018). Collagen–silica nanocomposites as dermal dressings preventing infection in vivo. Mater. Sci. Eng. C–Mater. Biol. Appl..

[124] Tao G., Wang Y.J., Cai R., Chang H.P., Song K., Zuo H., Zhao P., Xia Q.Y., He H.W. (2019). Design and performance of sericin/poly (vinyl alcohol) hydrogel as a drug delivery carrier for potential wound dressing application. Mater. Sci. Eng. C–Mater. Biol. Appl..

[125] Zhai M., Xu Y., Zhou B., Jing W. (2018). Keratin–chitosan/n–ZnO nanocomposite hydrogel for antimicrobial treatment of burn wound healing: Characterization and biomedical application. J. Photochem. Photobiol. B: Biol..

[126] Peppas N.A., Hilt J.Z., Khademhosseini A., Langer R. (2006). Hydrogels in Biology and Medicine: From Molecular Principles to Bionanotechnology. Adv. Mater..

[127] Goergen N., Wojcik M., Drescher S., Pinnapireddy S.R., Brussler J., Bakowsky U., Jedelska J. (2019). The Use of Artificial Gel Forming Bolalipids as Novel Formulations in Antimicrobial and Antifungal Therapy. Pharmaceutics.

[128] Langer R., Peppas N.A. (2003). Advances in Biomaterials, Drug Delivery, and Bionanotechnology. AICHE J..

[129] Kamoun E.A., Kenawy E.-R.S., Chen X. (2017). A review on polymeric hydrogel membranes for wound dressing applications: PVA–based hydrogel dressings. J. Adv. Res..

[130] Bonacucina G., Cespi M., Mencarelli G., Giorgioni G., Palmieri G. (2011). Thermosensitive Self–Assembling Block Copolymers as Drug Delivery Systems. Polymers.

[131] GhavamiNejad A., Park C.H., Kim C.S. (2016). In situ synthesis of antimicrobial silver nanoparticles within antifouling zwitterionic hydrogels by catecholic redox chemistry for wound healing application. Biomacromolecules.

[132] Fang H., Wang J.H., Li L., Xu L.Q., Wu Y.X., Wang Y., Fei X., Tian J., Li Y. (2019). A novel high–strength poly (ionic liquid)/PVA hydrogel dressing for antibacterial applications. Chem. Eng. J..

[133] Michailidou G., Christodoulou E., Nanaki S., Barmpalexis P., Karavas E., Vergkizi-Nikolakaki S., Bikiaris D.N. (2019). Super–hydrophilic and high strength polymeric foam dressings of modified chitosan blends for topical wound delivery of chloramphenicol. Carbohydr. Polym..

[134] Grip J., Engstad R.E., Skjæveland I., Skalko-Basnet N., Holsæter A.M. (2017). Sprayable Carbopol hydrogel with soluble beta–1,3/1,6–glucan as an active ingredient for wound healing—Development and in–vivo evaluation. Eur. J. Pharm. Sci..

[135] Aksu N.B., Yozgatli V., Okur M.E., Ayla S., Yoltas A., Okur N.U. (2019). Preparation and evaluation of QbD based fusidic acid loaded in situ gel formulations for burn wound treatment. J. Drug Deliv. Sci. Technol..

[136] Arafa M.G., El-Kased R.F., Elmazar M.M. (2018). Thermoresponsive gels containing gold nanoparticles as smart antibacterial and wound healing agents. Sci. Rep..

[137] Khan A., Xu M., Wang T.J., You C.G., Wang X.G., Ren H.T., Zhou H.W., Khan A., Han C.M., Li P. (2019). Catechol cross–linked antimicrobial peptide hydrogels prevent multidrug–resistant Acinetobacter baumannii infection in burn wounds. Biosci. Rep..

[138] Nnamani P.O., Kenechukwu F.C., Anugwolu C.L., Attama A.A. (2014). Evaluation of hydrogels based on poloxamer 407 and polyacrylic acids for enhanced topical activity of gentamicin against susceptible infections. Trop. J. Pharm. Res..

[139] Oryan A., Jalili M., Kamali A., Nikahval B. (2018). The concurrent use of probiotic microorganism and collagen hydrogel/scaffold enhances burn wound healing: An in vivo evaluation. Burns.

[140] Thi P.L., Lee Y., Thi T.T.H., Park K.M., Park K.D. (2018). Catechol–rich gelatin hydrogels in situ hybridizations with silver nanoparticle for enhanced antibacterial activity. Mater. Sci. Eng. C–Mater. Biol. Appl..

[141] George L., Bavya M.C., Rohan K.V., Srivastava R. (2017). A therapeutic polyelectrolyte–vitamin C nanoparticulate system in polyvinyl alcohol–alginate hydrogel: An approach to treat skin and soft tissue infections caused by Staphylococcus aureus. Colloids Surf. B. Biointerfaces.

[142] Khozemy E.E., Nasef S.M., Mahmoud G.A. (2018). Synthesis and characterization of antimicrobial nanocomposite hydrogel based on wheat flour and poly (vinyl alcohol) using γ–irradiation. Adv. Polym. Tech..

[143] Kumar A., Jaiswal M. (2016). Design and in vitro investigation of nanocomposite hydrogel based in situ spray dressing for chronic wounds and synthesis of silver nanoparticles using green chemistry. J. Appl. Polym. Sci..

[144] Ullah F., Othman M.B.H., Javed F., Ahmad Z., Akil H.M. (2015). Classification, processing and application of hydrogels: A review. Mater. Sci. Eng. C.

[145] Ono R.J., Lee A.L.Z., Chin W., Goh W.S., Lee A.Y.L., Yang Y.Y., Hedrick J.L. (2015). Biodegradable Block Copolyelectrolyte Hydrogels for Tunable Release of Therapeutics and Topical Antimicrobial Skin Treatment. ACS Macro Lett..

[146] Pertici V., Pin-Barre C., Rivera C., Pellegrino C., Laurin J., Gigmes D., Trimaille T. (2019). Degradable and injectable hydrogel for drug delivery in soft tissues. Biomacromolecules.

[147] dos Santos A.C.M., Akkari A.C.S., Ferreira I.R.S., Maruyama C.R., Pascoli M., Guilherme V.A., de Paula E., Fraceto L.F., de Lima R., Melo P. (2015). Poloxamer–based binary hydrogels for delivering tramadol hydrochloride: Sol–gel transition studies, dissolution–release kinetics, in vitro toxicity, and pharmacological evaluation. Int. J. Nanomedicine.

[148] Romić M.D., Klarić M.Š., Lovrić J., Pepić I., Cetina-Čižmek B., Filipović-Grčić J., Hafner A. (2016). Melatonin–loaded chitosan/Pluronic^®^ F127 microspheres as in situ forming hydrogel: An innovative antimicrobial wound dressing. Eur. J. Pharm. Biopharm..

[149] Wang C., Wang M., Xu T., Zhang X., Lin C., Gao W., Xu H., Lei B., Mao C. (2019). Engineering Bioactive Self–Healing Antibacterial Exosomes Hydrogel for Promoting Chronic Diabetic Wound Healing and Complete Skin Regeneration. Theranostics.

[150] Ali N.H., Amin M.C.I.M., Ng S.-F. (2019). Sodium carboxymethyl cellulose hydrogels containing reduced graphene oxide (rGO) as a functional antibiofilm wound dressing. J. Biomater. Sci. Polym. Ed..

[151] Capanema N.S.V., Mansur A.A.P., de Jesus A.C., Carvalho S.M., de Oliveira L.C., Mansur H.S. (2018). Superabsorbent crosslinked carboxymethyl cellulose–PEG hydrogels for potential wound dressing applications. Int. J. Biol. Macromol..

[152] Namazi H., Rakhshaei R., Hamishehkar H., Kafil H.S. (2016). Antibiotic loaded carboxymethylcellulose/MCM–41 nanocomposite hydrogel films as potential wound dressing. Int. J. Biol. Macromol..

[153] Ng S.-F., Jumaat N. (2014). Carboxymethyl cellulose wafers containing antimicrobials: A modern drug delivery system for wound infections. Eur. J. Pharm. Sci..

[154] Rakhshaei R., Namazi H. (2017). A potential bioactive wound dressing based on carboxymethyl cellulose/ZnO impregnated MCM–41 nanocomposite hydrogel. Mater. Sci. Eng. C.

[155] Yadollahi M., Gholamali I., Namazi H., Aghazadeh M. (2015). Synthesis and characterization of antibacterial carboxymethyl cellulose/ZnO nanocomposite hydrogels. Int. J. Biol. Macromol..

[156] Ahmed S., Ikram S. (2016). Chitosan Based Scaffolds and Their Applications in Wound Healing. Achiev. Life Sci..

[157] Liang Y., Zhao X., Hu T., Han Y., Guo B. (2019). Mussel–inspired, antibacterial, conductive, antioxidant, injectable composite hydrogel wound dressing to promote the regeneration of infected skin. J. Colloid. Interface Sci..

[158] Qu J., Zhao X., Liang Y., Zhang T., Ma P.X., Guo B. (2018). Antibacterial adhesive injectable hydrogels with rapid self–healing, extensibility and compressibility as wound dressing for joints skin wound healing. Biomaterials.

[159] Sung J.H., Hwang M.-R., Kim J.O., Lee J.H., Kim Y.I., Kim J.H., Chang S.W., Jin S.G., Kim J.A., Lyoo W.S. (2010). Gel characterisation and in vivo evaluation of minocycline–loaded wound dressing with enhanced wound healing using polyvinyl alcohol and chitosan. Int. J. Pharm..

[160] Hwang M.-R., Kim J.O., Lee J.H., Kim Y.I., Kim J.H., Chang S.W., Jin S.G., Kim J.A., Lyoo W.S., Han S.S. (2010). Gentamicin–loaded wound dressing with polyvinyl alcohol/dextran hydrogel: Gel characterization and in vivo healing evaluation. AAPS PharmSciTech.

[161] Kouchak M., Ameri A., Naseri B., Kargar Boldaji S. (2014). Chitosan and polyvinyl alcohol composite films containing nitrofurazone: Preparation and evaluation. Iran. J. Basic Med. Sci..

[162] Davis S.C., Li J., Gil J., Head C., Valdes J., Glinos G.D., Solis M., Higa A., Pastar I. (2019). Preclinical evaluation of a novel silver gelling fiber dressing on Pseudomonas aeruginosa in a porcine wound infection model. Wound Repair Regen..

[163] Patil P.P., Meshram J.V., Bohara R.A., Nanaware S.G., Pawar S.H. (2018). ZnO nanoparticle–embedded silk fibroin–polyvinyl alcohol composite film: A potential dressing material for infected wounds. New J. Chem..

[164] Yang S., Yang Y., Cui S., Feng Z., Du Y., Song Z., Tong Y., Yang L., Wang Z., Zeng H. (2018). Chitosan–polyvinyl alcohol nanoscale liquid film–forming system facilitates MRSA–infected wound healing by enhancing antibacterial and antibiofilm properties. Int. J. Nanomedicine.

[165] Huh A.J., Kwon Y.J. (2011). “Nanoantibiotics”: A new paradigm for treating infectious diseases using nanomaterials in the antibiotics resistant era. J. Controlled Release.

[166] Klouda L., Mikos A.G. (2008). Thermoresponsive hydrogels in biomedical applications. Eur. J. Pharm. Biopharm..

[167] Ricci E.J., Lunardi L.O., Nanclares D.M.A., Marchetti J.M. (2005). Sustained release of lidocaine from Poloxamer 407 gels. Int. J. Pharm..

[168] Becherer T., Vieira Nascimento M., Sindram J., Noeske P.-L.M., Wei Q., Haag R., Grunwald I. (2015). Fast and easily applicable glycerol–based spray coating. Prog. Org. Coat..

[169] Li M., Chen J., Shi M.T., Zhang H.L., Ma P.X., Guo B.L. (2019). Electroactive anti–oxidant polyurethane elastomers with shape memory property as non–adherent wound dressing to enhance wound healing. Chem. Eng. J..

[170] Li P., Poon Y.F., Li W.F., Zhu H.Y., Yeap S.H., Cao Y., Qi X.B., Zhou C.C., Lamrani M., Beuerman R.W. (2011). A polycationic antimicrobial and biocompatible hydrogel with microbe membrane suctioning ability. Nature Materials.

[171] Xie Z., Aphale N.V., Kadapure T.D., Wadajkar A.S., Orr S., Gyawali D., Qian G., Nguyen K.T., Yang J. (2015). Design of antimicrobial peptides conjugated biodegradable citric acid derived hydrogels for wound healing. J. Biomed. Mater. Res. A.

[172] Garcia C., Gallardo A., López D., Elvira C., Azzahti A., Lopez-Martinez E., Cortajarena A.L., González-Henríquez C.M., Sarabia-Vallejos M.A., Rodríguez-Hernández J. (2018). Smart pH–Responsive Antimicrobial Hydrogel Scaffolds Prepared by Additive Manufacturing. ACS Appl. Bio Mater..

[173] Seetharaman S., Natesan S., Stowers R.S., Mullens C., Baer D.G., Suggs L.J., Christy R.J. (2011). A PEGylated fibrin–based wound dressing with antimicrobial and angiogenic activity. Acta Biomater..

[174] Dubey P., Packirisamy G. (2016). PEGylated Graphene Oxide based Nanocomposite grafted Chitosan/Polyvinyl alcohol Nanofiber as an Advanced Antibacterial Wound Dressing. RSC Advances.

[175] Gil J., Natesan S., Li J., Valdes J., Harding A., Solis M., Davis S.C., Christy R.J. (2017). A PEGylated fibrin hydrogel–based antimicrobial wound dressing controls infection without impeding wound healing. Int. Wound J..

[176] Akash M.S.H., Rehman K. (2015). Recent progress in biomedical applications of Pluronic (PF127): Pharmaceutical perspectives. J. Controlled Release.

[177] Gioffredi E., Boffito M., Calzone S., Giannitelli S.M., Rainer A., Trombetta M., Mozetic P., Chiono V. (2016). Pluronic F127 hydrogel characterization and biofabrication in cellularized constructs for tissue engineering applications. Procedia CIRP.

[178] Nascimento M.H.M., Franco M.K.K.D., Yokaichyia F., de Paula E., Lombello C.B., de Araujo D.R. (2018). Hyaluronic acid in Pluronic F–127/F–108 hydrogels for postoperative pain in arthroplasties: Influence on physico–chemical properties and structural requirements for sustained drug–release. Int. J. Biol. Macromol..

[179] Dumortier G., Grossiord J.L., Agnely F., Chaumeil J.C. (2006). A Review of Poloxamer 407 Pharmaceutical and Pharmacological Characteristics. Pharm. Res..

[180] Dewan M., Bhowmick B., Sarkar G., Rana D., Bain M.K., Bhowmik M., Chattopadhyay D. (2015). Effect of methyl cellulose on gelation behavior and drug release from poloxamer based ophthalmic formulations. Int. J. Biol. Macromol..

[181] Ip M., Lui S.L., Poon V.K.M., Lung I., Burd A. (2006). Antimicrobial activities of silver dressings: An in vitro comparison. J. Med. Microbiol..

[182] Kouser R., Vashist A., Zafaryab M., Rizvi M.A., Ahmad S. (2018). Na–montmorillonite–dispersed sustainable polymer nanocomposite hydrogel films for anticancer drug delivery. ACS Omega.

[183] Das A., Kumar A., Patil N.B., Viswanathan C., Ghosh D. (2015). Preparation and characterization of silver nanoparticle loaded amorphous hydrogel of carboxymethylcellulose for infected wounds. Carbohydr. Polym..

[184] Friedrich E.E., Sun L.T., Natesan S., Zamora D.O., Christy R.J., Washburn N.R. (2014). Effects of hyaluronic acid conjugation on anti–TNF–α inhibition of inflammation in burns. J. Biomed. Mater. Res. A.

[185] Mayol L., Quaglia F., Borzacchiello A., Ambrosio L., Rotonda M.I.L. (2008). A novel poloxamers/hyaluronic acid in situ forming hydrogel for drug delivery: Rheological, mucoadhesive and in vitro release properties. Eur. J. Pharm. Biopharm..

[186] Price R.D., Berry M.G., Navsaria H.A. (2007). Hyaluronic acid: The scientific and clinical evidence. J. Plast. Reconstr. Aesthet. Surg..

[187] Liu L., Liu Y., Li J., Du G., Chen J. (2011). Microbial production of hyaluronic acid: Current state, challenges, and perspectives. Microb. Cell Fact..

[188] Ibrahim N.I., Wong S.K., Mohamed I.N., Mohamed N., Chin K.Y., Ima-Nirwana S., Shuid A.N. (2018). Wound Healing Properties of Selected Natural Products. Int. J. Env. Res. Public Health.

[189] Contardi M., Russo D., Suarato G., Heredia-Guerrero J.A., Ceseracciu L., Penna I., Margaroli N., Summa M., Spano R., Tassistro G. (2019). Polyvinylpyrrolidone/hyaluronic acid–based bilayer constructs for sequential delivery of cutaneous antiseptic and antibiotic. Chem. Eng. J..

[190] Cho K.Y., Chung T.W., Kim B.C., Kim M.K., Lee J.H., Wee W.R., Cho C.S. (2003). Release of ciprofloxacin from poloxamer–graft–hyaluronic acid hydrogels in vitro. Int. J. Pharm..

[191] Yang K., Han Q., Chen B., Zheng Y., Zhang K., Li Q., Wang J. (2018). Antimicrobial hydrogels: Promising materials for medical application. Int. J. Nanomedicine.

[192] Chen M.M., Tian J., Liu Y., Cao H., Li R.Y., Wang J.H., Wu J.L., Zhang Q.Q. (2019). Dynamic covalent constructed self–healing hydrogel for sequential delivery of antibacterial agent and growth factor in wound healing. Chem. Eng. J..

[193] Cheung R.C.F., Ng T.B., Wong J.H., Chan W.Y. (2015). Chitosan: An update on potential biomedical and pharmaceutical applications. Mar. Drugs.

[194] Irwansyah I., Li Y.-Q., Shi W., Qi D., Leow W.R., Tang M.B.Y., Li S., Chen X. (2015). Gram–Positive Antimicrobial Activity of Amino Acid–Based Hydrogels. Adv. Mater..

[195] Veiga A.S., Schneider J.P. (2013). Antimicrobial hydrogels for the treatment of infection. Biopolymers.

[196] Ali A., Ahmed S. (2018). A review on chitosan and its nanocomposites in drug delivery. Int. J. Biol. Macromol..

[197] Ahmadi F., Oveisi Z., Samani S.M., Amoozgar Z. (2015). Chitosan based hydrogels: Characteristics and pharmaceutical applications. Res. Pharm. Sci..

[198] Helander I.M., Nurmiaho-Lassila E.L., Ahvenainen R., Rhoades J., Roller S. (2001). Chitosan disrupts the barrier properties of the outer membrane of Gram–negative bacteria. Int. J. Food Microbiol..

[199] Choi Y.H., Han H. (2018). Nanomedicines: Current status and future perspectives in aspect of drug delivery and pharmacokinetics. J. Pharm. Investig..

[200] Yuan T.T., DiGeorge Foushee A.M., Johnson M.C., Jockheck-Clark A.R., Stahl J.M. (2018). Development of Electrospun Chitosan–Polyethylene Oxide/Fibrinogen Biocomposite for Potential Wound Healing Applications. Nanoscale Res. Lett..

[201] Hasan N., Cao J., Lee J., Hlaing S.P., Oshi M.A., Naeem M., Ki M.H., Lee B.L., Jung Y., Yoo J.W. (2019). Bacteria–targeted clindamycin loaded polymeric nanoparticles: Effect of surface charge on nanoparticle adhesion to MRSA, antibacterial activity, and wound healing. Pharmaceutics.

[202] Khansari S., Duzyer S., Sinha-Ray S., Hockenberger A., Yarin A.L., Pourdeyhimi B. (2013). Two–Stage Desorption–Controlled Release of Fluorescent Dye and Vitamin from Solution–Blown and Electrospun Nanofiber Mats Containing Porogens. Mol. Pharm..

[203] Teo W.-E., He W., Ramakrishna S. (2006). Electrospun scaffold tailored for tissue–specific extracellular matrix. Biotechnol. J..

[204] Miguel S.P., Sequeira R.S., Moreira A.F., Cabral C.S.D., Mendonca A.G., Ferreira P., Correia I.J. (2019). An overview of electrospun membranes loaded with bioactive molecules for improving the wound healing process. Eur. J. Pharm. Biopharm..

[205] Sill T.J., von Recum H.A. (2008). Electrospinning: Applications in drug delivery and tissue engineering. Biomaterials.

[206] Shahriar S.M.S., Mondal J., Hasan M.N., Revuri V., Lee D.Y., Lee Y.-K. (2019). Electrospinning Nanofibers for Therapeutics Delivery. Nanomaterials (Basel, Switzerland).

[207] Tao B.L., Lin C.C., Deng Y.M., Yuan Z., Shen X.K., Chen M.W., He Y., Peng Z.H., Hu Y., Cai K.Y. (2019). Opper–nanoparticle–embedded hydrogel for killing bacteria and promoting wound healing with photothermal therapy. J. Mat. Chem. B.

